# Adsorption Behavior of Diclofenac on Polystyrene and Poly(butylene adipate-*co*-terephthalate) Microplastics: Influencing Factors and Adsorption Mechanism

**DOI:** 10.1021/acs.langmuir.3c01536

**Published:** 2023-08-15

**Authors:** Siqi Liang, Kangkang Wang, Kefu Wang, Tao Wang, Changyan Guo, Wei Wang, Jide Wang

**Affiliations:** † Key Laboratory of Oil and Gas Fine Chemicals, Ministry of Education & Xinjiang Uygur Autonomous Region, School of Chemical Engineering and Technology, Xinjiang University, Urumqi 830046, China; ‡ Department of Chemistry, University of Bergen, Bergen 5007, Norway; § Centre for Pharmacy, University of Bergen, Bergen 5020, Norway

## Abstract

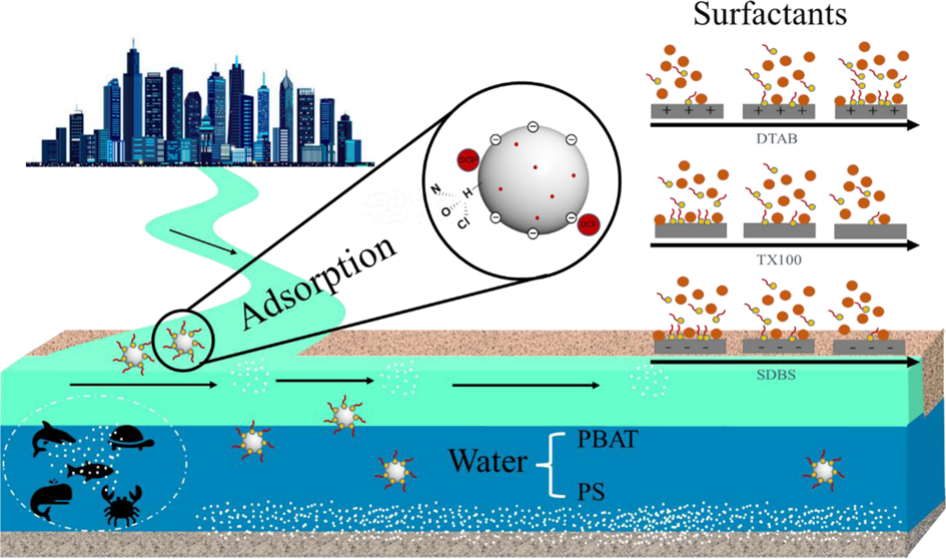

To unveil the intricacies surrounding the interaction between microplastics (MPs) and pollutants, diligent investigation is warranted to mitigate the environmental perils they pose. This exposition delves into the sorption behavior and mechanism of diclofenac sodium (DCF), a contaminant, upon two distinct materials: polystyrene (PS) and poly(butylene adipate-*co*-terephthalate) (PBAT). Experimental adsorption endeavors solidify the observation that the adsorption capacity of DCF onto the designated MPs amounts to *Q*_(PBAT)_ = 9.26 mg g^–1^ and *Q*_(PS)_ = 9.03 mg g^–1^, respectively. An exploration of the factors governing these discrepant adsorption phenomena elucidates the influence of MPs and DCF properties, environmental factors, as well as surfactants. Fitting procedures underscore the suitability of the pseudo-second-order kinetic and Freundlich models in capturing the intricacies of the DCF adsorption process onto MPs, corroborating the notion that the mentioned process is characterized by non-homogeneous chemisorption. Moreover, this inquiry unveils that the primary adsorption mechanisms of DCF upon MPs encompass electrostatic interaction, hydrogen bonding, and halo hydrogen bonding. An additional investigation concerns the impact of commonly encountered surfactants in aqueous environments on the adsorption of DCF onto MPs. The presence of surfactants elicits modifications in the surface charge properties of MPs, consequently influencing their adsorption efficacy vis-à-vis DCF.

## Introduction

Plastics, renowned for their characteristics of lightweight, cost-effectiveness, and ubiquity, find extensive employment in agriculture, industry, and the tapestry of human existence.^1,2^ Owing to their tenacity and chemical constancy, plastics endure in the environment for countless centuries, resisting decomposition with an unwavering resolve.^3^ This resilient accumulation of plastic detritus is incessantly fractured into minute fragments and particles by external forces, such as the ceaseless dance of wind, the churning caress of water, the unrelenting gaze of sunlight, and the unyielding pressure of biology. Once these plastic fragments reach dimensions below the threshold of 5 mm, they are christened microplastics (MPs).^4,5^ MPs, beyond emancipating the additives intricately infused within their manufacturing process, also display a proclivity for ensnaring neighboring contaminants, thereby imbricating the trajectories and destinies of these pollutants within the aqueous realm.^6^ During their ecological sojourn, these MPs serve as unwitting transporters, ferrying adsorbed organic pollutants into the domain of organisms, thereby begetting potential environmental conundrums and jeopardizing the sanctity of well-being. The vast array of organic pollutants engaging in a complex interplay with MPs has now captivated the attention of inquisitive minds, encompassing polycyclic aromatic hydrocarbons (PAHs), antibiotics, pesticides, and the entourage of bisphenol organics.^7−10^ Polystyrene (PS) reigns supreme as the most prevalent denizen of aquatic habitats, occupying the limelight amongst the pantheon of MPs.^11^ Additionally, the realm of biodegradable plastics, exemplified by polybutylene terephthalate adipate (PBAT), has begun its ascendance, permeating diverse applications, such as agricultural mulch, cling film, and the sturdy embrace of plastic bags.^12^ Delving into the intricate mechanisms of adsorption and interaction, bridging the chasm between degradable PBAT MPs and nondegradable PS MPs, and their effect on the entangled pollutants in the environment, shall furnish a foundation for impeding and mitigating the cataclysmic repercussions imposed by MPs upon our surroundings and the inhabitants they support.

Pharmaceuticals and personal care products (PPCPs) have emerged as organic pollutants that manifest themselves in natural water.^13^ The process of adsorption, which profoundly influences the mobility and ultimate destiny of PPCPs within the environment,^14^ assumes a position of paramount importance. Diclofenac (DCF), a common ingredient in pharmaceuticals and personal care products, finds widespread use in mitigating pain and inflammation in both human and animal subjects, yet upon its liberation into the environment, it metamorphoses into a representative organic pollutant.^15^ The global annual consumption of DCF is estimated to reach a staggering 940 tons.^16^ Thanks to its feeble biodegradability and elevated polarity, DCF withstands conventional municipal sewage systems with remarkable fortitude, resulting in meager removal rates ranging between 21 and 40% while lingering in water with ease.^17^ To further compound matters, DCF has earned its place in the EU Resolution 2015/495 watch list for surface waters, where its presence beyond concentrations exceeding 50 ng L^–1^ portends an environmental hazard,^14^ one that casts a looming shadow upon the well-being of aquatic organisms. It follows, therefore, that the scrutiny of DCF sorption by MPs and the projection of the ensuing transport dynamics of DCF-laden MPs within the environment represent indispensable endeavors.

Surfactants, ubiquitous in industrial and domestic realms, have been detected in wastewater at elevated concentrations. Notably, Narkis and Sun et al. have reported surfactant concentrations ranging from several hundred to several thousand mg L^–1^ in industrial wastewater.^18,19^ The structures of surfactants encompass copious oxygen-containing functional groups capable of modifying the sorption behavior and migration of pollutants on MPs.^20^ MPs, being hydrophobic organic pollutants, transform their physicochemical properties upon the introduction of surfactants, subsequently influencing their sorption characteristics in conjunction with other pollutants.^21−23^ Understanding the impact of chemical surfactants on the behavior of MPs within the environment assumes paramount significance. Alas, investigations into the sorption effects of MPs in the presence of chemical surfactants remain scarce. Furthermore, prior inquiries have primarily focused on the influence of non-degradable MPs on pollutant sorption while neglecting the repercussions of degradable MPs on pollutants. Consequently, the present study endeavors to employ DCF as a representative pollutant; PS and PBAT as model carriers to conduct a comparative examination of the dissimilar sorption capabilities of degradable and nondegradable plastics concerning organic pollutants. Additionally, the study encompasses an exploration of influential factors such as pH and salinity as environmental parameters. Moreover, the investigation systematically delves into the impact of MPs on the adsorption of DCF in the presence of surfactants.

## Materials and Methods

### Materials

Poly(butylene adipate-*co*-terephthalate) (PBAT) and polystyrene (PS) with particle sizes ranging within 75–150 μm were purchased from China Hengfa Plastic Technology Co. Ltd. Comprehensive information concerning the chemical structures and properties of PS and PBAT can be found in Table S1. Each batch of MPs underwent a thorough ultrasonic cleaning with deionized water, repeating the process three times for a duration of 5 min per cycle. Subsequently, the cleansed MPs were dried in an oven set at 50 °C for a period of 12 h and then cautiously stored for future use. Diclofenac (DCF), procured from China Aladdin Industries, Inc., played a central role in the experimentation, and its structure and properties are presented in Table S2. Other essential components employed in the study encompassed humic acid (HA) from China Beijing Domestic Technology Co., sodium hydroxide (NaOH), hydrochloric acid (HCl), and sodium chloride (NaCl) from China Aladdin Industries. The utilization of deionized water was consistent across all experimental procedures, with the purity of the unmentioned reagents adhering to analytical standards. The selected surfactants, namely, the cationic dodecyl trimethyl ammonium bromide (DTAB), anionic sodium dodecyl benzene sulfonate (SDBS), and nonionic Triton X-100 (TX100), were procured from Aladdin Industries, Inc.

### Characterization of MPs

To characterize the MPs, the samples were vacuum dried for at least 3 days before use. The surface morphology of PBAT and PS was characterized using scanning electron microscopy (SEM). Fourier transform infrared (FTIR) spectroscopy was used to study the changes in surface structure and functional groups of PBAT and PS before and after adsorption with the wavenumber in the range of 400–4000 cm^–1^. The ζ-potential was employed to determine the trend of the surface potential of MPs with pH. The contact angle was used to test the hydrophilicity of PBAT and PS. At the same time, the specific surface area analysis was used to test the specific surface area of PBAT and PS.

### Adsorption Experiments

The method of intermittent equilibrium was employed to assess the adsorption capacity of MPs on DCF. All adsorption experiments took place within 60 mL brown glass bottles containing a solution volume of 50 mL. The pH of the solution was carefully adjusted to 7.0 ± 0.2. The bottles were gently shaken at a temperature of 25 ± 1°C and a speed of 160 rpm. To prevent any potential interactions, the concentration of methanol was maintained below 0.1% (v/v), thereby avoiding cosolvent interference. The initial concentration of DCF stood at 20 mg L^–1^. Throughout the experiment, the mass of the MPs remained consistently at 10 mg. The kinetic experiment focused on monitoring time intervals ranging from 0 to 36 h (0, 0.5, 1, 3, 6, 9, 12, 24, 36 h) as key observation points. Based on the kinetic experiment, it was deduced that the adsorption equilibrium of MPs for DCF was achieved after 24 h, indicating a sufficient duration for reaching adsorption equilibrium. Subsequently, in the subsequent experiments, the samples were placed within a thermostatic oscillator for a period of 24 h to ensure equilibrium. During the adsorption isothermal experiment, DCF solutions with varying initial concentrations were prepared, ranging from 15 to 35 mg L^–1^. Additionally, 10 mg of PBAT and PS was introduced into 50 mL of DCF solution with different concentrations.

The influence of pH, humic acid, ionic strength, and surfactants on the adsorption of DCF by MPs was examined. To achieve precision, the pH of the solution was modulated using a solution of 0.1 mol·L^–1^ HCl and 0.1 mol·L^–1^ NaOH, thereby attaining a pH range of 3–9. The adsorption capacity of DCF onto MPs was measured across various pH values. The impact of diverse concentrations of humic acid (ranging from 0 to 20 mg L^–1^) on the adsorption properties of DCF was probed. Furthermore, the effect of ionic strength (with NaCl concentrations spanning from 0 to 0.60 mol L^–1^) on the adsorption of DCF by MPs was meticulously explored. Concentrations of surfactants ranged from 0 to 60 mg L^–1^. Concomitantly, control experiments were established, including a blank control devoid of plastic and a control with plastic but lacking pollutants. The change of pollutants mixed with aqueous solution for a long time was verified by a blank controlled experiment without MPs, and the change of MPs in aqueous solution was obtained by a controlled experiment containing MPs but without pollutants. These aforementioned experiments were conducted in triplicate for enhanced accuracy. When repeated experiments were carried out, each group of experiments is carried out in three groups at the same time to ensure that all experimental conditions are consistent with the original experiment to eliminate the influence of external conditions on the experiment.

## Results and Discussion

### Characterization of MPs

**Figure 1:**
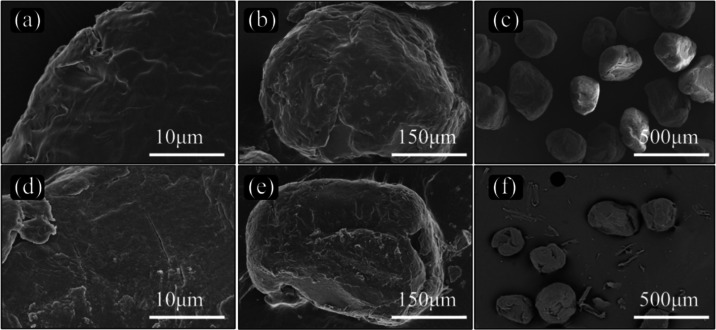
SEM images of PBAT (a–c) and PS (d–f).

The affinity of microplastics (MPs) toward pollutants is intricately related to their surface morphology and structure. To study this phenomenon, a thorough characterization of PBAT and PS was carried out via SEM. Figure 1a,d unveils the captivating surface morphology of PBAT and PS, unveiling a predominantly smooth exterior punctuated by delicate undulations. Notably, the disparity in surface roughness between PBAT and PS is marginal. Furthermore, both MPs exhibit a uniform particle size of approximately 150 μm, as discernible in Figure 1c,f.

**Figure 2:**
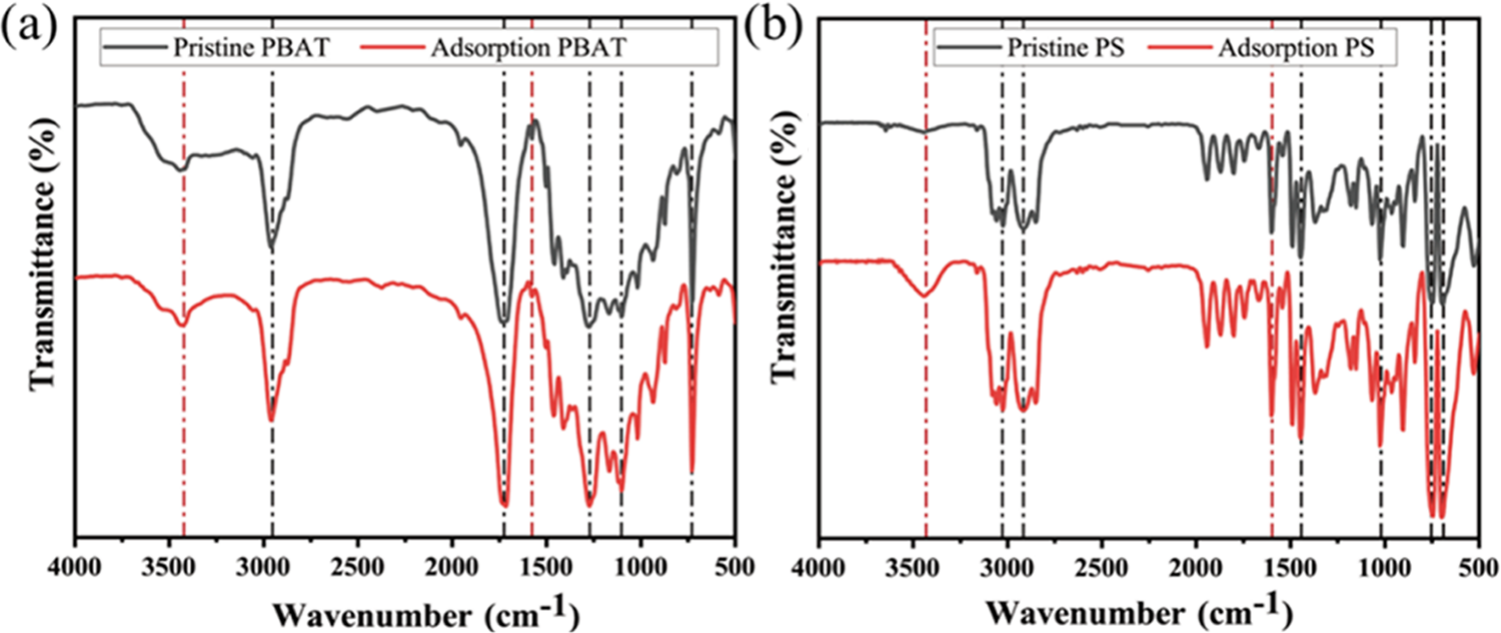
FTIR of PBAT (a) and PS (b) before and after adsorption.

Figure 2 shows the FTIR spectra, illustrating the targeted MPs before and following adsorption. It can be deduced that PBAT exhibits robust peak intensities at 1050, 1156, 1179, 1276, 1720, and 2960 cm^–1^, corresponding to O–C–O, C–O, primary hydroxyl (−CH_2_–OH), secondary hydroxyl (−CH–OH), C=O, and C–H stretching vibrations, respectively. Post adsorption, a shift in the hydroxyl functional group of PBAT is observed, moving from its original position at 3428–3438 cm^–1^. Additionally, the vibrational peak of the initial PBAT benzene ring at 1576 cm^–1^ undergoes a shift to 1579 cm^–1^ after adsorption. As for PS, it exhibits prominent peak intensities at 720, 750, 1490, 2916, and 3059 cm^–1^, corresponding to the stretching vibrations of C–H_3_, C–H, C–H, and C–H_2_, respectively. PS demonstrates a more intense peak at 1596 cm^–1^, which corresponds to the stretching and bending vibrations of C–H on the benzene ring backbone. The FTIR spectrum of PS reveals that the vibrational peak of the original PS benzene ring is located at 1596 cm^–1^, shifting to 1605 cm^–1^ after adsorption. Furthermore, a hydroxyl functional group emerges at 3448 cm^–1^ following adsorption, indicating the absorption of DCF through hydrogen bonding.^24^ In a similar study, Liu et al. detected a hydroxyl functional group at 3500 cm^–1^ in the FTIR spectra when ciprofloxacin was adsorbed with PS, suggesting hydrogen bonding as a plausible mechanism during the adsorption process.^25^ In conclusion, the hydroxyl group emerges as the primary functional group on the surface of MPs that undergo alteration before and after adsorption, with other functional groups also experiencing displacement. These observations underscore the significant role of hydrogen bonding and halo hydrogen bonding in the adsorption process.

**Figure 3:**
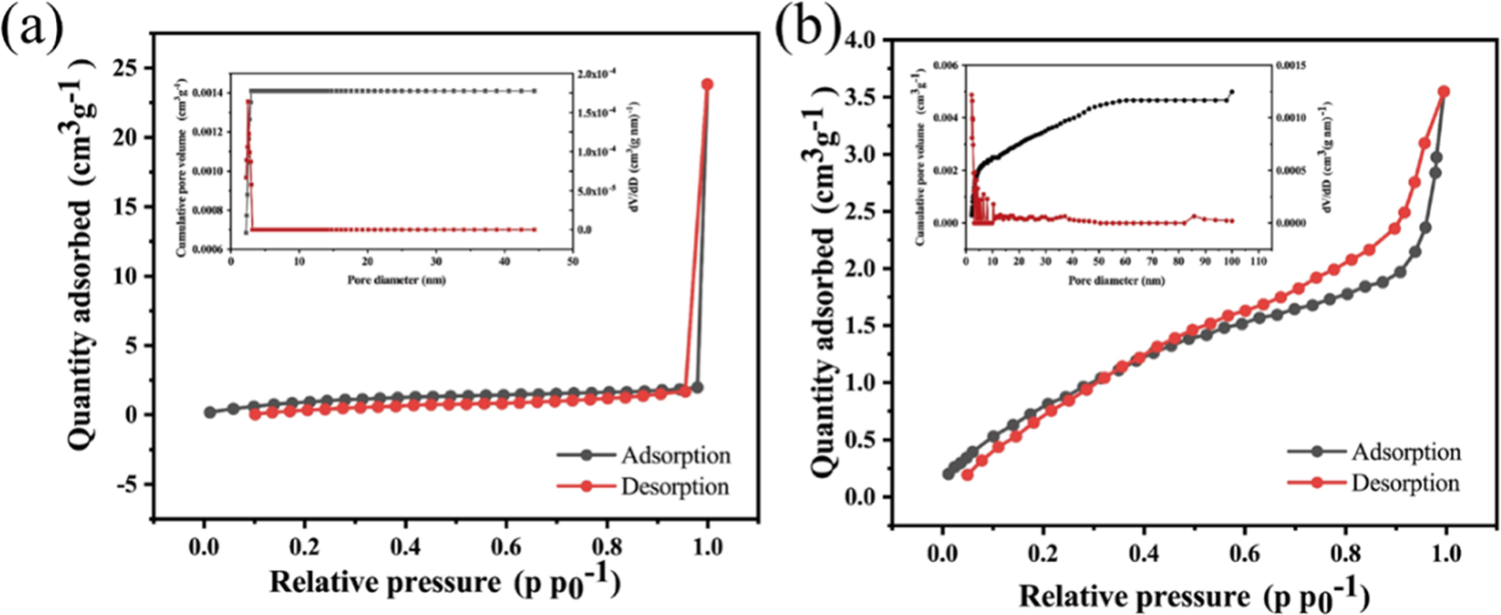
Nitrogen adsorption/desorption curves of PBAT (a) and PS (b).

Figure 3 shows the isothermal nitrogen adsorption/desorption curves for the targeted MPs, with the following results: specific surface area: PBAT = 4.191 m^2^ g^–1^ > PS = 3.652 m^2^ g^–1^. The difference in specific surface area between the two MPs is small, but the pore volume and pore size of PBAT are significantly larger than those of PS (pore volume: PBAT = 33.711 cm^3^ mg^–1^ > PS = 5.333 cm^3^ mg^–1^; pore size: PBAT = 8.683 nm > PS = 5.833 nm).

**Figure 4:**
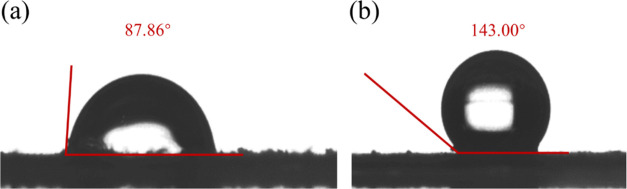
Contact angles of PBAT (a) and PS (b).

The contact angle unveils the hydrophilic properties of MPs, with smaller angles indicative of heightened hydrophilicity. As elucidated by the findings presented in Figure 4, the contact angle of PBAT measures 87.86°, evincing its inherent hydrophilic nature. Conversely, PS exhibits a contact angle of 143°, signifying its hydrophobic tendencies. It becomes apparent that PS surpasses PBAT in terms of hydrophobicity. This distinction arises primarily from the contrasting abundance of hydrophobic groups present on the surface of the MPs. Such observations align seamlessly with the distinctive structural characteristics inherent to each MP variant, as the PS surface hosts a greater number of hydrophobic groups than its PBAT counterpart, thus rendering it more hydrophobic.

### Adsorption Kinetics

**Figure 5:**
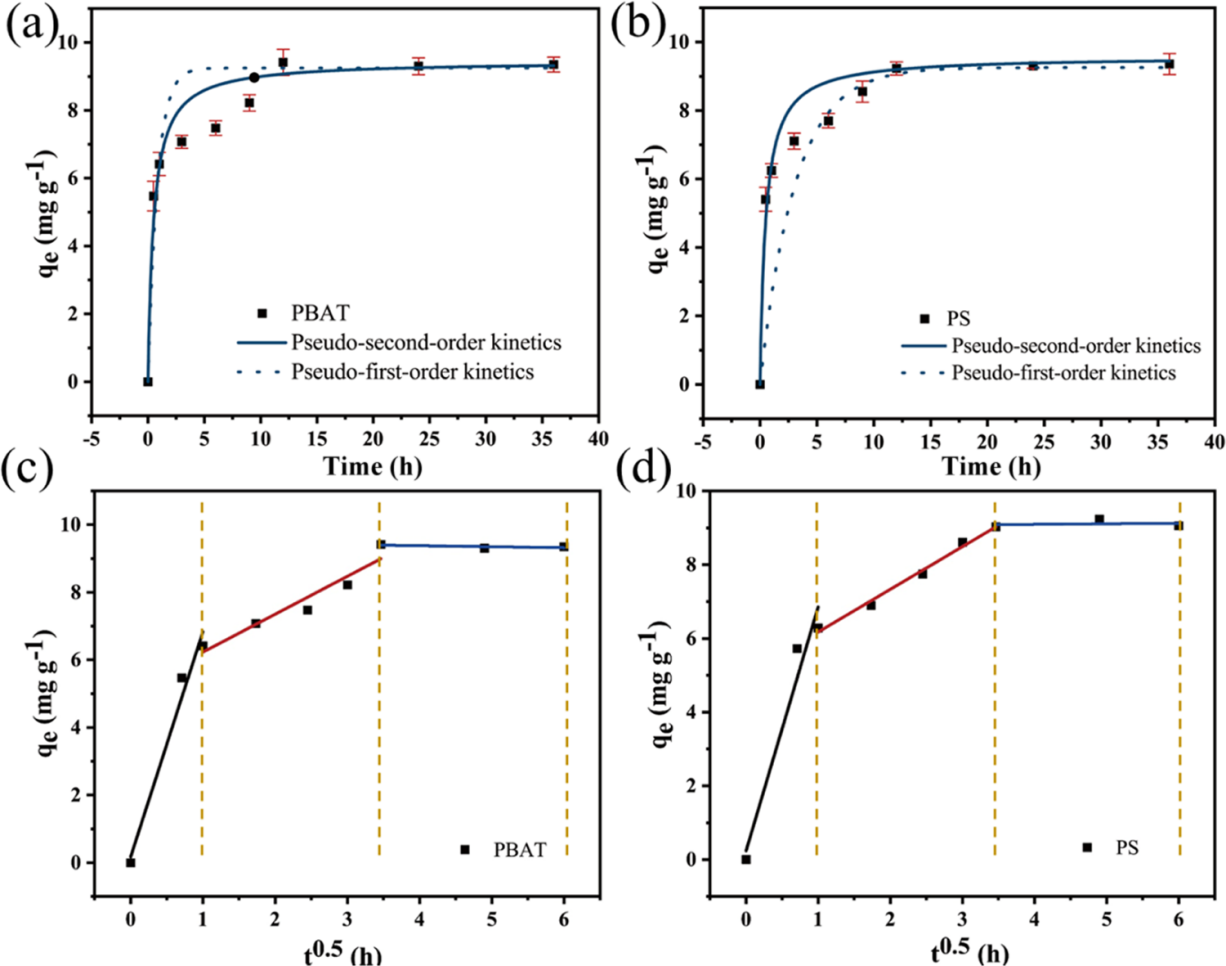
Pseudo-first-order kinetics and pseudo-second-order kinetics of DCF adsorption on PBAT (a) and PS (b) and intraparticle diffusion model on PBAT (c) and PS (d).

The adsorption kinetics find common employment in describing the temporal equilibrium of adsorption, the mechanism governing such adsorption, and the rate at which pollutants adhere to MPs.^26^ For PBAT and PS, the adsorption behavior of DCF has been examined through the lens of pseudo-first-order kinetics, pseudo-second-order kinetics, and intraparticle diffusion in Figure 5. Quantitatively, the actual quantities of DCF adsorbed onto the MPs were as follows: *Q*_(PBAT)_ = 9.26 mg g^–1^ and *Q*_(PS)_ = 9.03 mg g^–1^. The adsorption capacity of MPs remains inextricably linked to their inherent physicochemical properties. In the SEM images, the minute disparity in surface roughness between the two MPs becomes apparent. Theoretically, it follows that the greater the specific surface area and pore volume, the more pollutants an MP could accumulate. While the specific surface area between the selected MPs for this experimental endeavor only exhibits a minor discrepancy, with PBAT slightly surpassing PS, the pore volume of PBAT far surpasses that of PS. Thus, the infiltration mechanism, despite initial inclinations, does not emerge as the primary impetus behind the formidable adsorption of DCF onto PBAT and PS. Figure 5a,b and Table S3 lie the data of the adsorption kinetics of DCF onto MPs. We identify the pseudo-second-order kinetic model’s superior *R*^2^ value, better than that of its pseudo-first-order kinetic counterpart for both MPs, while also aligning more closely with the experimental adsorption quantities. This unambiguous revelation corroborates the supposition that the adsorption of DCF onto PS and PBAT predominantly adheres to the pseudo-second-order kinetic model, indicative of the prevalence of chemisorption.^26^

Without intersecting the origin, the adsorption fitting plot for internal particle diffusion reveals a nonlinear association between *q_t_* and *t*^0.5^. This observation suggests that the internal diffusion process does not singularly constitute the rate-determining step, and the adsorption rate may also be influenced by the external diffusion process. The adsorption process can be demarcated into three stages. The first stage entails surface adsorption, wherein DCF occupies the active sites on the exterior surface of MPs through hydrophobic partitioning, covalent bonding forces, and van der Waals forces. The second stage encompasses intraparticle diffusion, where DCF surmounts the resistance imposed by the outer liquid film and permeates toward the inner surface of MPs. The third stage represents the quasi-equilibrium adsorption stage.^27^ The constant diffusion rates in the three stages adhere to the following order: *K*_1_ > *K*_2_ > *K*_3_. This observation can likewise be verified through the intraparticle diffusion model illustrated in Figure 5c,d. The initial phase of the experiment exhibits a rapid surge in adsorption, constituting a swift adsorption process characterized by the steepest slope during this time. Subsequently, over a duration of 4 h, the adsorption rate gradually levels off until reaching 12 h. This phase corresponds to a slow adsorption process that approaches dynamic equilibrium near the 12 h mark, as evidenced by a slope nearing zero. Following the initial 12 h period, adsorption essentially achieves dynamic equilibrium. To summarize, the adsorption of DCF onto the two MPs manifests as a chemisorption process governed by both internal and external diffusion.

### Adsorption Isotherms

**Figure 6:**
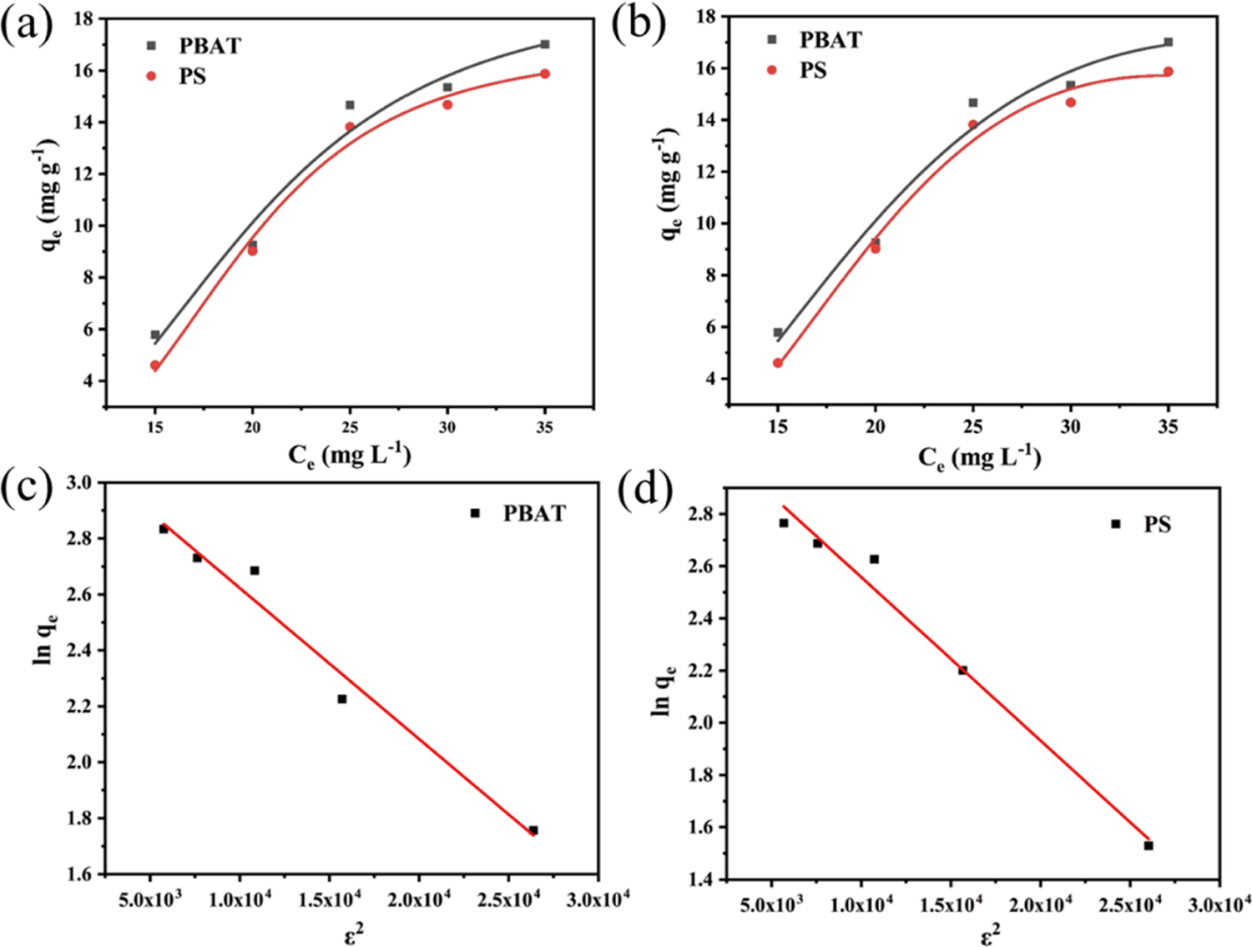
Langmuir model (a) and Freundlich model (b) of DCF adsorption on MPs, D-R model of DCF adsorption on PBAT (c), and D-R model of DCF adsorption on PS (d).

The interaction between pollutant and adsorbent at adsorption equilibrium can be anticipated by adsorption isotherm models. The results of the isothermal adsorption experiment further verified that the 24 h adsorption experiment was sufficient to achieve the equilibrium. Within the realm of isothermal sorption, three frequently employed models, namely, Langmuir, Freundlich, and D-R, were employed to fit the isothermal sorption data (Figure 6). The sorption isotherms of DCF on both MPs exhibited nonlinearity, suggesting that the sorption capacity of the MPs was contingent upon the concentration and distribution effects of DCF.^24^ As presented in Table S5, the Freundlich isothermal adsorption model yielded superior *R*^2^ values compared to the Langmuir and D-R adsorption models. The Freundlich isotherm model represents an empirical approach to non-homogeneous adsorption,^28,29^ while D-R is employed to elucidate adsorption mechanisms on non-homogeneous surfaces, assuming a multilayer nature encompassing van der Waals forces. Hence, non-homogeneous adsorption influences the adsorption of DCF by MPs, while van der Waals forces contribute to the process as well. Simultaneously, following the findings of the D-R model fitting, the average free energy of adsorption surpasses 16 kJ mol^–1^. This observation signifies that the chemisorption of DCF by MPs predominates as the underlying process, thereby providing further validation to the adsorption kinetics results.

### Influence of Environmental Factors on Sorption

#### Effect of pH on Adsorption

**Figure 7:**
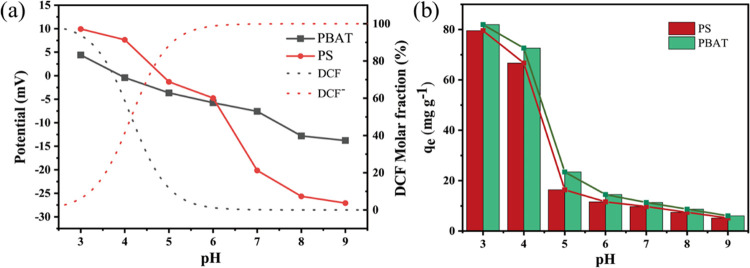
ζ-Potential of MPs at different pH (a) and effect of solution pH on adsorption (b).

The pH of the solution exerts an influence on the surface charge of MPs and alters the form of DCF present within the solution, thereby impacting the adsorption behavior of DCF onto MPs. These results are presented in Figure 7b, where the adsorption capacity of DCF on MPs undergoes a rapid decline as pH levels increase. MPs exhibit a pronounced adsorption capacity for DCF under acidic conditions, while the adsorption amount gradually diminishes as pH increases. Figure 7a depicts the progression of the charge properties of the MPs’ surface as pH levels fluctuate. It becomes apparent that the MPs’ surface manifests distinct charge properties across varying pH values, with both exhibiting a positive charge at low pH. As the solution’s pH increases, the positive charge weakens, and the MPs’ surface assumes a negative charge upon reaching the zero-point charge. While pH remains below the equivalence point, the MPs’ surface remains positively charged, and the degree of positive charge intensifies as pH decreases. Conversely, when the pH exceeds the DCF’s equivalence point, DCF transitions gradually from its nonionized form to its ionized form. Consequently, the proportion of the nonionized form diminishes as pH increases, and a concurrent rise occurs in the proportion of the anionic form.^30^ Moreover, as evidenced in Figure 7b, the abrupt decline in DCF adsorption by MPs when pH shifts from 4 to 5 arises from the conversion of DCF from its molecular form to its anionic form, transpiring concurrently with the MPs acquiring a negative charge on their surface. This sudden escalation in the electrostatic pulse between the anionic DCF^–^ and negatively charged MPs results in reduced adsorption of DCF by MPs when the solution’s pH > 4.

When the pH < 4, the high adsorption of DCF by both MPs is mainly controlled by hydrogen bonding, which is consistent with the FTIR results. In contrast, when pH > 4, electrostatic repulsion dominates, resulting in lower adsorption of DCF by MPs, which is also consistent with the experimental results. In summary, electrostatic interactions dominate the adsorption of DCF by MPs, while hydrogen bonding and halogen–hydrogen bonding are also the mechanisms of the adsorption process.^31^

#### Effect of HA and Ionic Strength on Adsorption

**Figure 8:**
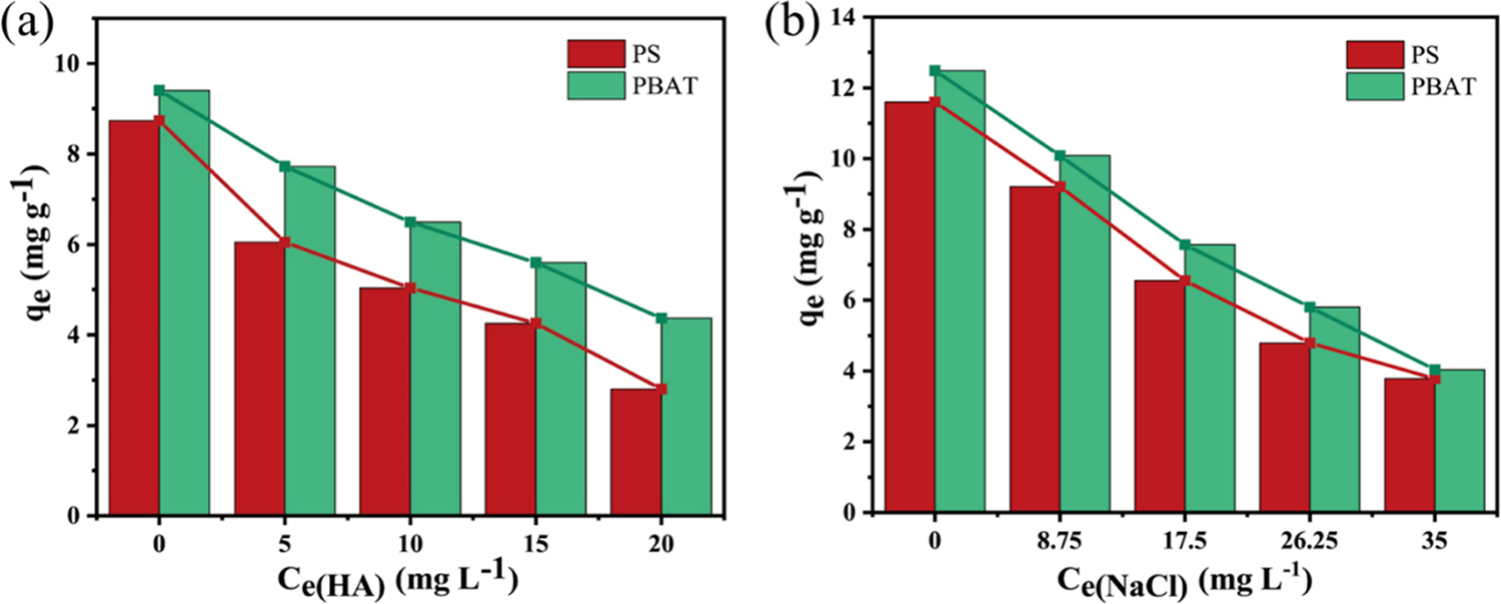
Effect of humic acid strength (a) and salinity (b) on adsorption.

Humic acid (HA) is ubiquitous in the natural environment and exists in almost all water environments and plants.^32^ In the water environment, there is inevitable contact with MPs, which affects the interaction between MPs and pollutants. The influence of varying concentrations of HA on the adsorption of DCF onto PBAT and PS was meticulously examined, as illustrated in Figure 8a. As the HA concentration amplifies, the quantity of DCF adsorbed onto both MPs progressively diminishes. This outcome arises from the profusion of functional groups within HA molecules, allowing them to engage with MPs and organic substances, thereby impacting the adsorption efficacy of DCF upon MPs. Furthermore, the organic macromolecules constituting HA might lay claim to adsorption sites on the surface of MPs, replacing DCF’s occupancy. Alternatively, they may engage DCF through intricate hydrophobic interactions, culminating in curtailment of DCF’s adsorptive capacity upon MPs. Thus, HA’s presence can potentially hinder the dual jeopardy presented by DCF and MPs. Ai et al. have also substantiated that the presence of HA inhibits the adsorption progression of As(III) through positional resistance and competitive adsorption with As(III).^33^

NaCl is widely present in rivers, lakes, and oceans. Moreover, it has an important influence on the adsorption of organic pollutants by MPs. The average salinity of seawater is 35‰ (36.2 mg L^–1^).^9^ Accordingly, ionic strengths ranging from 0 to 0.60 mol L^–1^ (0, 8.75, 17.5, 26.25, 35 mg L^–1^) were selected to emulate the sorption processes of DCF and MPs across salinities ranging from freshwater to seawater. As depicted in Figure 8b, a discernible reduction in the adsorption of DCF onto MPs ensues as salinity escalates from 0 to 0.60 mol L^–1^. This decline in adsorption capacity arises due to the augmentation of ionic strength, potentially attributable to several factors: (1) heightened ionic strength prompts increased competition between Na^+^ ions and contaminants for adsorption sites, facilitated by ion exchange;^34^ (2) augmented ionic strength fosters the agglomeration of MPs, thereby reducing the availability of surface adsorption sites;^35^ and (3) elevated ionic strength induces a solubilization effect attributable to salt, whereby the introduction of NaCl impedes the mass transfer process from aqueous to solid phases by augmenting solution viscosity and density.^36^ Chen has also corroborated that salinity curtails the adsorption of pollutants by MPs.^26^ To summarize, MPs may face augmented perils within freshwater realms, where selecting organic pollutants evince a greater propensity for absorption onto MPs compared to seawater.

#### Effect of Surfactant Incorporation on Adsorption

**Figure 9:**
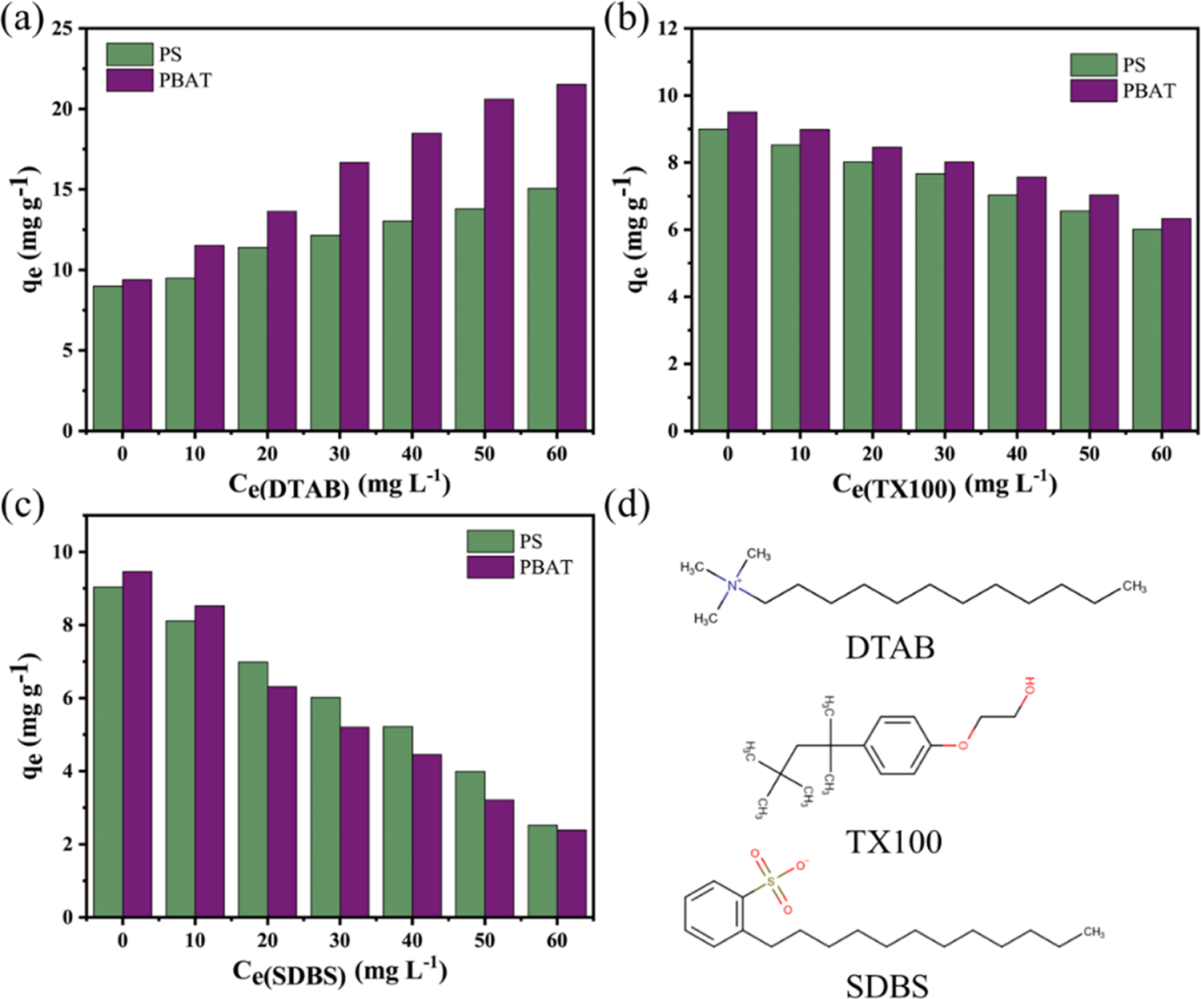
Effect of surfactant on adsorption: (a) DTAB, (b) TX100, (c) SDBS, and (d) structures of surfactants.

The addition of surfactants can change the surface tension of the solution and the charge present on the surface of the MPs. In this particular investigation, three surfactants were chosen: the cationic surfactant dodecyl trimethyl ammonium bromide (DTAB), the nonionic surfactant Triton X-100 (TX100), and the anionic surfactant sodium dodecyl benzene sulfonate (SDBS). Figure 9 explicitly demonstrates the discernible influence of surfactants on the adsorption of DCF (an acronym representing a substance of interest) onto PBAT and PS. Notably, the introduction of DTAB augments the adsorption of DCF by MPs, whereas the converse holds for SDBS and TX100. When immersed in aqueous solutions, the presence of a surfactant engenders alterations in the surface charge of the MPs.^20^ The adsorption process materializes within a neutral milieu, given that DCF manifests itself in an anionic form (DCF^–^). Upon introduction of an anionic surfactant, it binds to the MPs, thereby accentuating the negative charge on their surface. Consequently, the escalated electrostatic repulsion impedes the adsorption of DCF by the MPs. Conversely, upon the addition of cationic surfactants, the positive charge on the MPs’ surface intensifies, instigating an electrostatic attraction that facilitates heightened adsorption of DCF by the MPs. In contrast, within the TX100/MP system, the molecules of TX100 adhere to the MP surface through carbon chains and hydrogen bonds, thereby competing with DCF for adsorption sites. Consequently, the adsorption of DCF by PBAT and PS is diminished. The suppression of adsorption by anionic surfactants surpasses that of TX100, thus once again substantiating the predominant role of electrostatic interaction within the adsorption process.

### Adsorption Mechanism

The adsorption process is influenced by the hydrophobic and structural properties of MPs, as depicted in Figure 4. PS exhibits a contact angle of 143°, indicating its hydrophobic nature. Conversely, PBAT possesses a contact angle of 87.86°, signifying its hydrophilic character. Meanwhile, DCF, a hydrophobic pollutant, is confirmed to possess a log *K*_ow_ value of 4.51 (Table S2). Interestingly, sorption experiments revealed that DCF exhibited slightly higher sorption on PBAT compared to PS, implying that hydrophobic effects alone were not accountable for the heightened sorption of DCF on both MPs.

The FTIR spectra provide additional insights. The broad characteristic peaks observed in the range of 3500–3100 cm^–1^ are attributed to the vibrations of hydroxyl or carboxyl groups and intermolecular hydrogen bonding.^37^ It is worth noting that PBAT contains a greater number of oxygen functional groups, thereby reinforcing the presence of stronger hydrogen bonding and halo hydrogen bonding interactions between DCF and PBAT. This observation emphasizes the importance of hydrogen bonding as a significant mechanism in the adsorption process. Additionally, surface morphology analyses through SEM and BET experiments reveal that both MPs possess rough surfaces, with PBAT exhibiting a substantially larger pore volume compared to PS. Nevertheless, the disparity in adsorption between PBAT and PS is not significant, indicating that the filling mechanism does not represent the primary adsorption mechanism.

**Figure 10:**
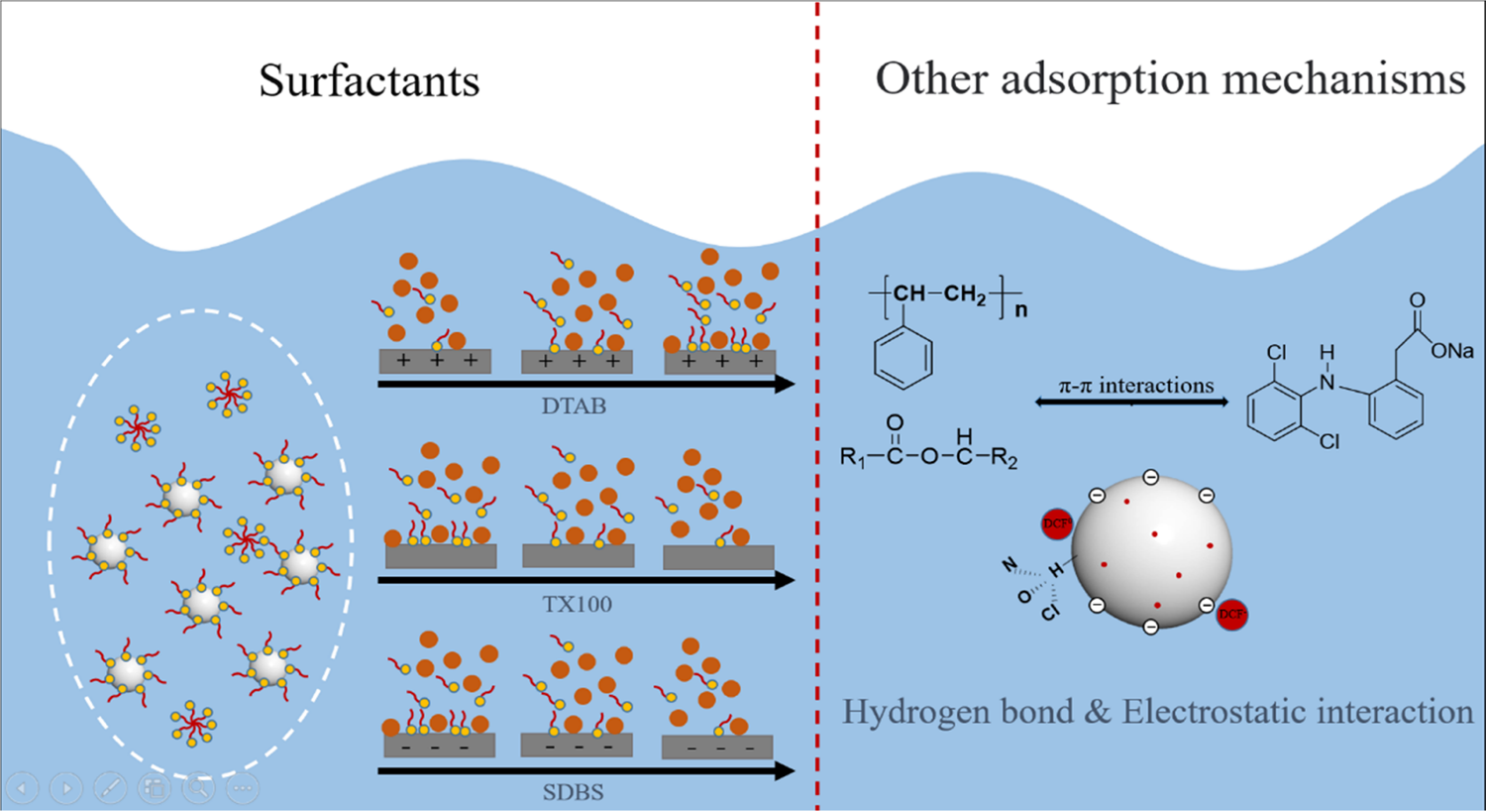
Schematic diagram of the adsorption mechanism of DCF on PBAT and PS.

The adsorption process adheres to the pseudo-second-order kinetic model and the Freundlich isothermal adsorption model. Moreover, the internal diffusion model demonstrates that the fitted lines do not intersect the origin, suggesting that the adsorption of DCF by MPs follows a nonuniform chemisorption process regulated by internal and external diffusion. Drawing upon the aforementioned analysis, Figure 10 depicts the adsorption mechanism of PBAT and PS on DCF. When the solution maintains a low pH (pH < pH_PZC_), both PBAT and PS carry a positive surface charge, while DCF exists in a molecular form. Consequently, the primary adsorption process is governed by the formation of hydrogen bonding and halo hydrogen bonding between the nitrogen and oxygen atoms on DCF, and the hydrogen atoms on MP chlorine atoms. Conversely, at higher pH values (pH > pH_PZC_), the surface charge of the MPs turns negative, and DCF assumes an anionic state. As a result, the adsorption of MPs onto DCF is diminished due to the presence of electrostatic repulsion. Figure 7a visually illustrates that the adsorption is considerably greater at low pH than at high pH and experiences a sudden decline between pH 4 and 5. Furthermore, the introduction of surfactants serves as further evidence that electrostatic interactions substantially influence the adsorption of MPs onto DCF. In conclusion, electrostatic interactions, hydrogen bonding, and halo hydrogen bonding emerge as the predominant forces in the adsorption of DCF by PBAT and PS.

## Conclusions

We studied the adsorption behavior and mechanism of DCF upon nondegradable PS and PBAT. The adsorption capacity of DCF by the intended MPs was found to be as follows: *Q*_(PBAT)_ (9.30 mg g^–1^) > *Q*_(PS)_ (9.21 mg g^–1^). The attainment of adsorption equilibrium for DCF on both PBAT and PS transpired after approximately 24 h. The fitting of the adsorption data aligns more coherently with pseudo-second-order kinetics and the Freundlich isothermal adsorption processes, thereby affirming a non-homogeneous chemisorption course. An experimental assessment of environmental factors demonstrated that DCF adsorption on both MPs was markedly influenced by pH, ionic strength, and humic acid. Acidic conditions were observed to be more favorable for the adsorption of DCF by MPs, while increased ionic strength and humic acid content impeded the adsorption process. Additionally, the inclusion of surfactants exhibited an impact on the adsorption performance of MPs concerning DCF. These findings collectively unveil that the adsorption process is chiefly regulated by electrostatic interaction, hydrogen bonding, and halo hydrogen bonding. With a focus on comprehending the interaction mechanism between MPs and the organic pollutant DCF, this paper presents a plausible adsorption mechanism, thereby providing a theoretical foundation for studying the interaction between MPs and pollutants.

## Supporting Information Available

Structure and properties of MPs, structure and properties of DCF; and model parameters for adsorption of pollutants by MPs (PDF)
