# Supplementary material for: Adsorption Behavior of Diclofenac on Polystyrene and Poly(butylene adipate-co-terephthalate) Microplastics: Influencing Factors and Adsorption Mechanism
Source: Langmuir. 2023 Aug 15;39(34):12216–25. doi: 10.1021/acs.langmuir.3c01536 (PMC10469442; doi:10.1021/acs.langmuir.3c01536)
Supplement: Supplementary file 1 — la3c01536_si_001.pdf [file la3c01536_si_001.pdf]

Supplementary Data

Adsorption behavior of diclofenac on polystyrene and poly(butylene adipate-co-terephthalate) microplastics: Influencing factors and adsorption mechanism

Siqi Liang [a], Kangkang Wang [a], Kefu Wang [a], Tao Wang [a], Changyan Guo [a,*], Wei Wang [b,*], Jide Wang [a,*]

[a] *Key Laboratory of Oil and Gas Fine Chemicals, Ministry of Education & Xinjiang Uygur Autonomous Region, School of chemical engineering and technology, Xinjiang University, Urumqi, China. E-mail: Changyan Guo: gcyslw@xju.edu.cn, Jide Wang: awangjd@sina.cn*

[b] *Department of Chemistry, University of Bergen, Bergen, 5007, Norway, E-mail: Wei Wang: wei.wang@uib.no*

[c] *Centre for Pharmacy, University of Bergen, Bergen, 5020, Norway*

Support information brief contents:

1. Table S1: Structure and properties of the polymers of MPs.

2. Table S2: Structure and properties of the DCF.

3. Table S3: Pseudo-first-order kinetics and pseudo-second-order kinetics parameters for PBAT and PS adsorption of DCF.

4. Table S4: Parameters of the intra-particle diffusion model for DCF adsorption on PBAT and PS.

5. Table S5: Adsorption isotherm parameters of MPs for DCF.

Table S1 Structure and properties of the polymers of MPs

MPs	Molecule Structure	Polarity	PZC	m.p.
PBAT	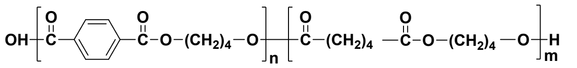	polar	3.92	130℃
PS	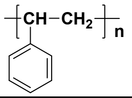	non-polar	4.86	166℃

Table S2 Structure and properties of the DCF

Compound	Structure	log K_{ow}	pKa	Water Solubility	Molecular Weight
Diclofenac (DCF)	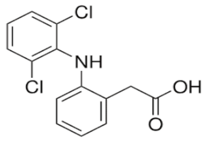	4.51	4.15	2.37 mg/L (25 ºC)	296.15 g/mol

Table S3 Pseudo-first-order kinetics and pseudo-second-order kinetics parameters for PBAT and PS adsorption of DCF

MPs	Pseudo-first-order kinetics			Pseudo-second-order kinetics		
	q_e (mg g^{-1})	K_1(h^{-1})	R^2	q_e (mg g^{-1})	K_2 (g (mg h)$^{-1}$)	R^2
PBAT	8.461±0.375	0.177±0.075	0.904	8.958±0.325	2.5222±0.665	0.958
PS	8.340±0.353	0.162±0.070	0.912	8.861±0.285	2.743±0.664	0.966

Table S4 Parameters of the intra-particle diffusion model for DCF adsorption on PBAT and PS

MPs	First stage			Second stage			Third stage		
	C_1	K_1	R_1^2	C_2	K_2	R_2^2	C_3	K_3	R_3^2
PBAT	0.236	6.617	0.957	5.015	1.156	0.990	9.033	0.015	0.031
PS	0.173	6.663	0.977	5.109	1.121	0.921	9.494	0.076	0.429

Table S5 Adsorption isotherm parameters of MPs for DCF

MPs	Langmuir			Freundlich			D-R		
	q_m (mg g^{-1})	K_L (L mg^{-1})	R^2	K_F (mg g^{-1})	$1/n$	R^2	q_m (mg g^{-1})	E (kJ mol^{-1})	R^2
PBAT	18.97	0.0022	0.953	21.04	1.106	0.977	3.163	96.23	0.966
PS	16.83	0.0002	0.981	29.24	1.152	0.990	3.184	89.35	0.983
